# PhenoDB, GeneMatcher and VariantMatcher, tools for analysis and sharing of sequence data

**DOI:** 10.1186/s13023-021-01916-z

**Published:** 2021-08-18

**Authors:** Elizabeth Wohler, Renan Martin, Sean Griffith, Eliete da S. Rodrigues, Corina Antonescu, Jennifer E. Posey, Zeynep Coban-Akdemir, Shalini N. Jhangiani, Kimberly F. Doheny, James R. Lupski, David Valle, Ada Hamosh, Nara Sobreira

**Affiliations:** 1 Department of Genetic Medicine, Johns Hopkins University School of Medicine, Baltimore, MD USA; 2 Center for Inherited Disease Research - CIDR, Johns Hopkins School of Medicine, Baltimore, MD USA; 3 Department of Molecular and Human Genetics, Baylor College of Medicine, Houston, TX USA; 4 Human Genome Sequencing Center, Baylor College of Medicine, Houston, TX USA; 5 Department of Pediatrics, Baylor College of Medicine, Houston, TX USA; 6 Texas Children’s Hospital, Houston, TX USA

**Keywords:** PhenoDB, GeneMatcher, VariantMatcher, Data sharing, Genomic data

## Abstract

**Background:**

With the advent of whole exome (ES) and genome sequencing (GS) as tools for disease gene discovery, rare variant filtering, prioritization and data sharing have become essential components of the search for disease genes and variants potentially contributing to disease phenotypes. The computational storage, data manipulation, and bioinformatic interpretation of thousands to millions of variants identified in ES and GS, respectively, is a challenging task. To aid in that endeavor, we constructed PhenoDB, GeneMatcher and VariantMatcher.

**Results:**

PhenoDB is an accessible, freely available, web-based platform that allows users to store, share, analyze and interpret their patients’ phenotypes and variants from ES/GS data. GeneMatcher is accessible to all stakeholders as a web-based tool developed to connect individuals (researchers, clinicians, health care providers and patients) around the globe with interest in the same gene(s), variant(s) or phenotype(s). Finally, VariantMatcher was developed to enable public sharing of variant-level data and phenotypic information from individuals sequenced as part of multiple disease gene discovery projects. Here we provide updates on PhenoDB and GeneMatcher applications and implementation and introduce VariantMatcher.

**Conclusion:**

Each of these tools has facilitated worldwide data sharing and data analysis and improved our ability to connect genes to phenotypic traits. Further development of these platforms will expand variant analysis, interpretation, novel disease-gene discovery and facilitate functional annotation of the human genome for clinical genomics implementation and the precision medicine initiative.

**Supplementary Information:**

The online version contains supplementary material available at 10.1186/s13023-021-01916-z.

## Background

In 2012, we developed the computational tool PhenoDB with the vision of producing a web-based tool that allows users naïve to bioinformatics to collect, store, analyze, and share phenotypic (including family structure and family phenotype data) and genomic sequence data. In 2013, we established GeneMatcher as a web-based platform to connect individuals (researchers, clinicians, health care providers, patients and families) and other organizations and stakeholders (e.g. pharmaceutical industry) around the globe with interest in the same genes, variants or phenotypes. In 2019, we developed VariantMatcher, a web application to publicly share variant-level data from ES/GS coupled with clinically observed phenotypic information from individuals sequenced as part of multiple disease gene discovery efforts. These tools have facilitated variant prioritization and data sharing for investigators around the world and greatly accelerated the disease gene discovery process.

## Results

### PhenoDB

**Fig. 1:**
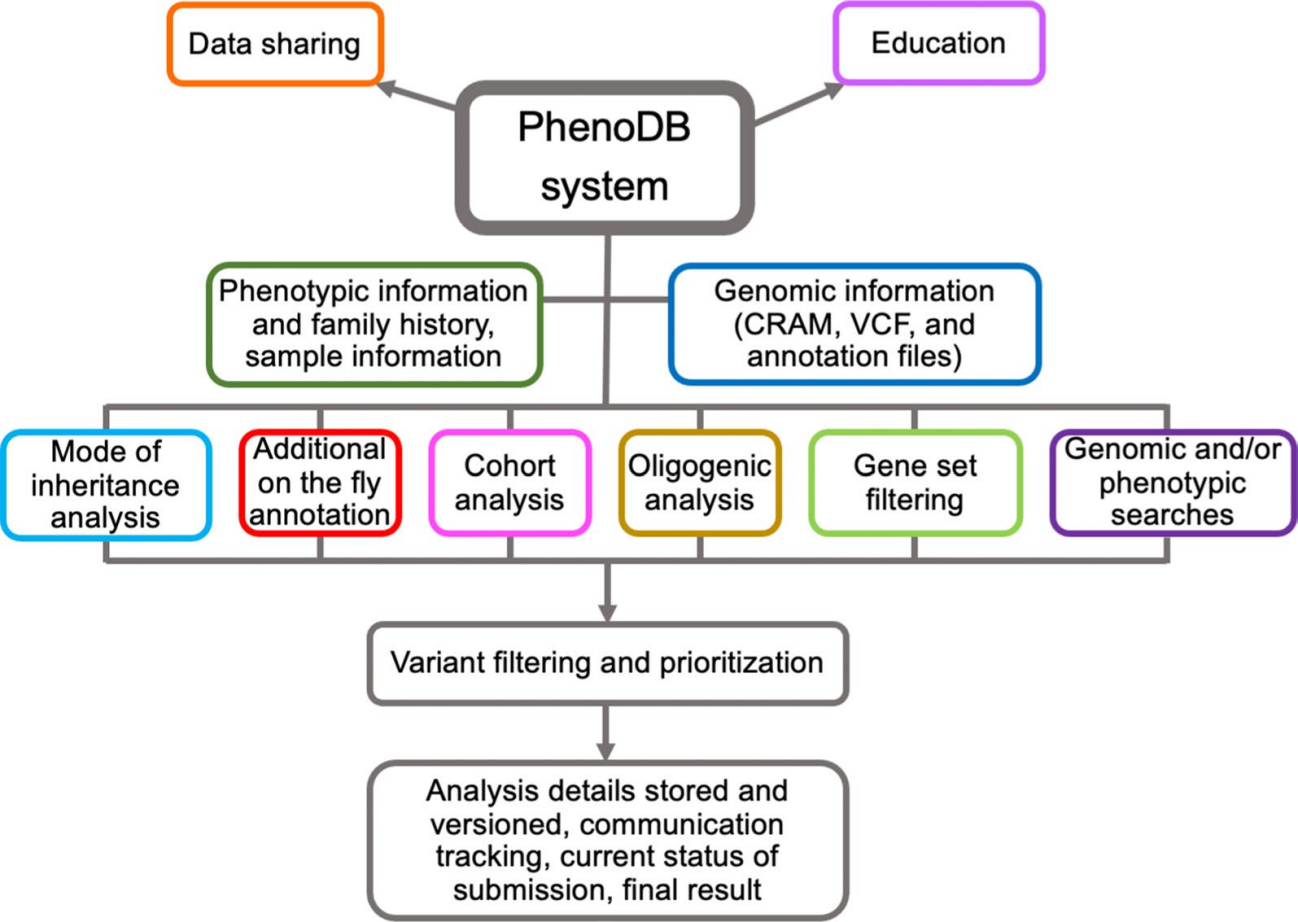
PhenoDB, a web-based database for collecting, storing, analyzing, and sharing phenotypic and genomic data

PhenoDB is a web-based system for collecting, storing, managing and analyzing phenotypic, clinical, family, and sequencing information using a combination of structured and unstructured fields to capture a standard data set while supporting flexible data types such as patient images, prior clinical diagnostic testing reports, family structure (e.g. pedigree) information, and consenting information (Fig. 1) [1]. It includes the ES and/or GS Sample Tracking and Variant Analysis Modules to assist in the process of variant filtering and candidate gene prioritization strategies [2]. The ‘front-end’ user interface was written using Python and the Django web application framework. Phenotypic data is persisted in a MySQL database, while larger-scale genomic data is stored and accessed via a SOLR server running on Tomcat. This resource houses a collection of structured data such as: VCF files and ANNOVAR annotated files; MySQL queries and complex comparison queries of the phenotypic data; the ability to scale-as-required by adding more nodes; a secure, compliant-ready environment; and granular control of data access privileges. This software enables the warehousing and analysis of large volumes of genomic and phenotypic data while providing rapid sample-level access on the order of a few seconds.

### Update on PhenoDB capabilities

#### Variant analysis module

PhenoDB stores VCF and annotated files for each sequenced individual. When these files are loaded into PhenoDB, automated autosomal dominant (monoallelic), autosomal recessive homozygous and compound heterozygous (biallelic variation at a locus) analyses are performed for variant prioritization and as a means to explore potential hypothesized Mendelian models. By default, these automated analyses compare the annotated files from all sequenced family members and select the rare (minor allele frequency [MAF] set by user, default < 1% in 1000 Genome, Exome Variant Server, ExAC and gnomAD), coding (missense, nonsense, stop-loss, synonymous affecting splicing, and indels) and splice site variants that segregate by each mode of inheritance. The analysis results are stored and can be downloaded as a tab delimited text or Excel file. The analysis result file also contains variant and gene annotations added by PhenoDB (e.g. RVIS score [Residual Variation Intolerance Score] [3], OMIM phenotype and mode of inheritance [4], MGI phenotype [5], GTEx expression [6], STRING interaction [7], Gene Ontology [8, 9], PubMed gene link, ClinVar classification and variant link [10], UniProt gene link [11], Gene Imprint information [12], GWAS Catalog phenotypes [13], TraP score [14], VarSome variant link [15], among others not available with the ANNOVAR annotation). Additional modes of inheritance can be investigated: X-linked recessive, X-linked dominant, maternal imprinting and paternal imprinting. The user can modify the parameters of the analysis (e.g. Refgene gene location, variant MAF, variant depth coverage, RVIS percentile and coordinate restriction) as desired.

#### Phenotypic information and variant analysis integration

As phenotypic features for each affected individual are added, PhenoDB automatically performs a diagnostic search in OMIM and presents a list of the 20 most likely clinical differential diagnoses. Additionally, when an analysis is performed, PhenoDB compares the genes in the analysis result file with the genes known to cause any of the 20 diagnoses listed as most likely based on the OMIM search. These matches are shown in a specific column in the analysis result file called OMIM Matching Phenotypes to support rapid identification of potentially pathogenic variants in established “disease genes”. Additionally, once phenotypic features are added for a specific individual, other individuals with a similar phenotype can be identified in the database. This search is based on the matching algorithm described by Wang et al. [16].

#### Cohort analysis

In this analysis, the user can compare results from multiple probands by searching for genes mutated in multiple individuals in a studied cohort. This analysis is performed by comparing analysis result files for distinct, unrelated probands (e.g. one may query for any 3 individuals in a cohort of 10 families who have variants in the same gene in the autosomal dominant analysis result files).

#### Oligogenic analysis

In this analysis, the user can compare results from multiple probands and ask the question: who are the individuals with the same 2 or more genes mutated? (e.g. individuals X and Y both have rare functional coding variants in genes A, B and C).

#### Filter function

Any ANNOVAR or analysis result file can be custom filtered. For example, an analysis result file can be filtered to retain only the variants in the genes in the ACMG secondary findings list [17] or the variants in a selected gene list defined by the user. Any ANNOVAR file or analysis result file can also be filtered to select variants in an existing list of genes associated with a phenotypic series from OMIM. The user can also select for variants in genes whose protein products interact (first-, second-, or third-order interactions) with those of other genes of interest, this analysis is based on STRING information [10].

#### Genomic search

The user can search for all the individuals in PhenoDB with a variant in a specific gene or with a specific variant (using genomic location with or without the nucleotide change). The search can be based on the ANNOVAR files, analysis result files, or final result files (a list of final candidate causative variants and genes selected for a specific proband). If searching among the analysis result files, the user can also select a specific mode of Mendelian inheritance (e.g.: autosomal dominant de novo analysis result files with variants in *PTEN*). Additionally, the search can be narrowed by adding features. For example, the user can identify all the autosomal recessive homozygous files with biallelic variants in *SPATA5* in probands with microcephaly.

#### Phenotypic search

In the PhenoDB Submission Search section, individuals with the same specific clinical feature(s) can be identified. The search can be based on an OMIM disease number (omim.org) or phenotypic features. There is an option to identify individuals who share all or any of the features of interest. Additionally, each proband entered in the database can be matched with other probands in the database by a phenotype comparison that identifies the individuals with a specified coefficient overlap (e.g.: all individuals with an at least 80% overlap of phenotypic features) [16].

#### Data sharing

Access to the phenotypic and genomic information related to a specific group of families can be granted to a specific group of researchers who can then independently analyze the data from all the probands, perform multiple phenotypic or genomic searches and share the results amongst themselves using the PhenoDB platform. For example, samples from 51 probands with VACTERL association from multiple unrelated researchers were sequenced by different Centers. ES was performed at the Center for Inherited Disease Research (CIDR, Johns Hopkins University (JHU) or the Human Genome Sequencing Center (HGSC, Baylor College of Medicine). Individual VCF files were independently created in the sequencing centers and uploaded to Mendelian Genomics PhenoDB (PhenoDB hosted on local JHU server). In Mendelian Genomics PhenoDB, VCF files were converted to ANNOVAR files and autosomal dominant and recessive inheritance models were tested. Now, as a multi-PI collaboration, the whole phenotypic and genomic dataset is being jointly analyzed by different methods across collaborating groups that have access to the same data in the Mendelian Genomics PhenoDB database. In this manner, PhenoDB supports ease of data sharing and collaborative analysis phenotypic and genomic datasets. We are using the same approach for the analysis of other phenotypes such as cleft lip and palate, PHACE syndrome (OMIM 606,519), Ollier disease (OMIM 166,000), Maffucci syndrome (OMIM 614,569) and others.

### Future implementations

The PhenoDB source code has been downloaded in 143 Institutions and based on user feedback and evolving analysis needs, new implementations are continually made. Further deployable developments that are underway include: (1) storage of BAM/CRAM files; and, (2) variant analysis using files generated on hg38. Planned improvements include: (1) integration of the IGV (Integrative Genomics Viewer) functionality for visualization of the sequencing reads; (2) integration of OpenCRAVAT, as a result visualization option [18]; (3) a new user interface for analysis results; (4) CNV and mitochondrial sequencing analysis; and (5) placement of the PhenoDB analysis engine in a Docker container.

The core analytic functions of PhenoDB are modules in Python 2 and Java 11, intertwined with a MySQL backend along with a SOLR searching platform. These analysis modules are orthogonal to the front-end web components which will facilitate the placement of the PhenoDB analysis engine in a Docker container. This anticipated change has multiple advantages: easier integration with other workflow management tools, such as AnVIL and CAVATIVA; increased scalability across local and cloud resources, as we can spin up multiple container-based analyses; and the ability to independently modify and upgrade the PhenoDB front end.

### PhenoDB implementation and experience

**Fig. 2:**
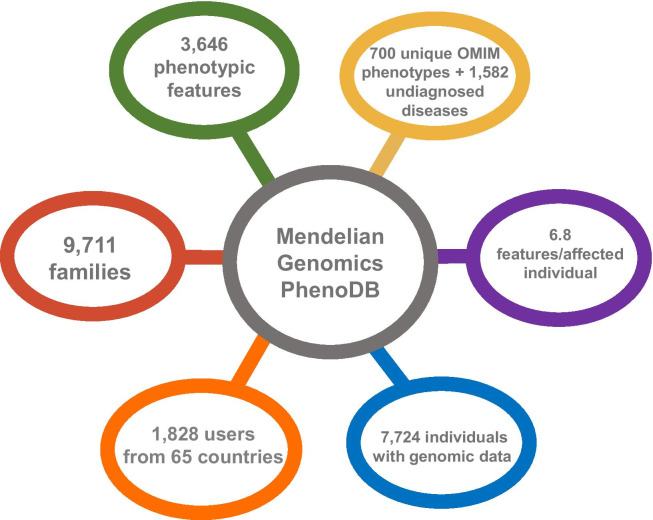
Mendelian Genomics PhenoDB phenotypic and genomic data

Mendelian Genomics, the specific PhenoDB implemented on the JHU server, stores deep phenotypic data based on 3646 PhenoDB feature terms (mapped to the Human Phenotype Ontology [19]) from 37,712 individuals in 9711 families and genomic data from 5973 affected and 1751 unaffected individuals. There are 700 unique OMIM phenotypes described in the Mendelian Genomics PhenoDB in addition to 1582 phenotypes that submitters were unable to match to a known OMIM phenotype. On average there are 6.8 phenotypic features per affected individual in the database. Different data access permissions are granted to different users based on patient/family informed consent and user role by the system administrators, but all available data, phenotypic and genomic, are fully searchable by administrators. Currently the Mendelian Genomics PhenoDB database has 1,828 users (collaborators from multiple sequencing projects) from 65 countries facilitating data sharing and analysis access world-wide (Fig. 2).

All data stored in Mendelian Genomics PhenoDB resides on a system owned and monitored by Johns Hopkins University. This system undergoes daily security scans and the back end can be accessed only while on an active VPN session and via two factor authentication. Access to the back end of the system is limited to required staff, including system administrators and software developers, all of whom undergo yearly training for IT Security and the safety and security of PHI data. Users of Mendelian Genomics must register for an account and only have access to their own submissions unless additional access permissions are granted by the system administrators.

An API connects Mendelian Genomics PhenoDB to GeneMatcher and VariantMatcher. When a final result file is created with the strongest candidate causative variants and genes for a specific proband, the candidate genes with no OMIM phenotypes (maximum of 10 genes), the variants, and the phenotypic information can be automatically sent to GeneMatcher to identify other investigators or clinicians with a shared interest in a gene(s). On a monthly basis, these genes are sent from GeneMatcher to other Matchmaker Exchange (MME) nodes.

As of 1 June 2020, 768 candidate genes had been sent from Mendelian Genomics PhenoDB to GeneMatcher and the other MME nodes. Additionally, as of 1 December 2020, 6,151 Mendelian Genomics PhenoDB individuals were consented for phenotypic and genotypic matching through VariantMatcher.

### GeneMatcher

**Fig. 3:**
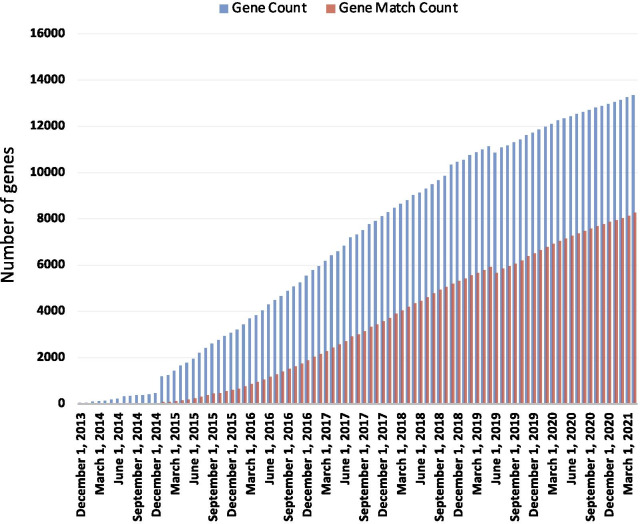
GeneMatcher Statistics as of 1 April 2021. Blue bars—total number of genes in GeneMatcher; red bars—number of genes in GeneMatcher that matched

GeneMatcher is a web-based tool constructed and implemented to connect individuals (researchers, clinicians, health care providers and patients) around the world with interest in the same genes, variants or phenotypes [2, 20]. As of 1 April 2021, there were 52,239 submissions to GeneMatcher of 13,337 unique genes by 11,044 submitters from 93 countries. Matches in GeneMatcher have been documented to support > 421 peer reviewed publications describing > 320 novel disease genes (Fig. 3).

### An analysis of the data stored in GeneMatcher through 1 June 2020

From September 2013 to 1 June 2020, 9,512 investigators from 90 countries utilized GeneMatcher. These users submitted 12,414 genes to GeneMatcher generating matches for 7,684 genes (~ 62%). As of 1 June 2020, there were 4,730 genes in GeneMatcher that never matched (~ 38%) and 2,822 genes that were submitted ≥ 5 (~ 23%) times. Of the total genes entered, 3,087 were already associated with a disease phenotype in OMIM (~ 25%).

#### Genes with only one entry in GeneMatcher (never matched)

For 4730 genes in GeneMatcher (38.1%) no match was obtained as of 1 June 2020; 2757 of these had been in GeneMatcher for more than one year. Only 9 of the 4730 genes (0.2%) were among the 154 disease-causing candidate genes selected by Hansen et al. [21]. Hansen and colleagues [21] used a genocentric analysis based on in silico predictions to analyze WES data from 18,696 individuals and identified 154 genes harboring variants suspected to cause Mendelian disorders. Among the 4,730 unmatched genes in GeneMatcher, 3,873 were not associated with a phenotype in OMIM (81.9%). We used the gene-based score tool Residual Variation Intolerance Score (RVIS) and the gnomAD constraint metrics, Z score and pLI score (as per Hansen et al. [21]), to evaluate the 3,873 genes.

**Fig. 4:**
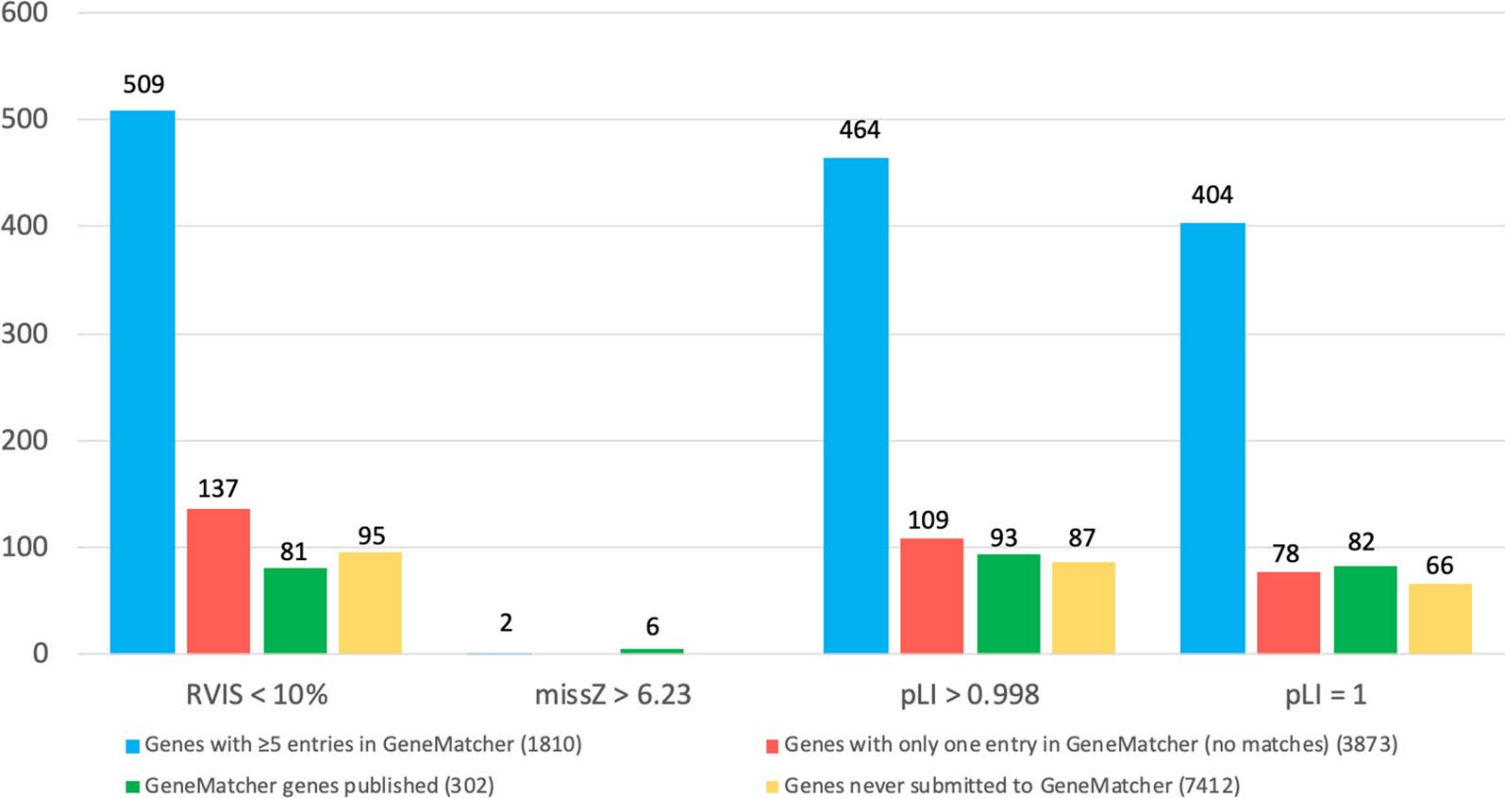
Number of genes in 4 GeneMatcher categories further grouped by in silico scores. pLI > 0.998 is used as in Hansen et al. [21] and the genes listed under pLI > 0.998 include the genes with pLI = 1

-  ~ 3.5% of the 3,873 genes had a RVIS percentile score < 10%, indicating that these 137 genes were within the top 10% of the genes most constrained for variation (Fig. 4, red bar).
- None of them had a missense Z score > 6.23 (Fig. 4, red bar).
- And, 109 genes had a pLI score > 0.998 (~ 3%) (Fig. 4, red bar). Of these, 78 genes had a pLI score = 1 (2%) (Fig. 4, red bar).

Among the 4730 unmatched genes, 857 are associated with a phenotype in OMIM, for 186 (~ 22%) of these the gene-phenotype relationship was established after 1 September 2013, and 57 (~ 31%) of the 186 were only associated with a phenotype in OMIM after being deposited in GeneMatcher.

#### Genes with ≥ 5 entries in GeneMatcher

Of the 12,414 genes submitted to GeneMatcher, 2,822 genes (~ 23%) had ≥ 5 entries, each submitted by unrelated investigators. 109 of the 2,822 genes (~ 3.9%) were identified by Hansen et al. [21] as one of their 154 candidate disease-causing genes. 1810 of the 2822 (~ 64%) were not associated with a phenotype in OMIM.

-  ~ 28% of the 1,810 genes had a RVIS percentile score < 10% (Fig. 4, blue bar).
- Only two genes had a missense Z > 6.23 (0.11%) (Fig. 4, blue bar).
- And, 464 genes had a pLI score > 0.998 (~ 26%) (Fig. 4, blue bar). Of these 464 genes, 404 had a pLI score = 1 (~ 22%) (Fig. 4, blue bar).

Of the 1012 genes that were associated with a phenotype in OMIM, the majority, 567 (56%), were associated with a phenotype after 1 September 2013. Of the 567 genes, 445 were associated with a phenotype in OMIM after being deposited in GeneMatcher (~ 78%).

#### Genes never submitted to GeneMatcher

Of the 19,133 protein-coding genes listed by the GENCODE version 19 of the haploid reference human genome, 8544 were not yet submitted to GeneMatcher (44.7%); 7412 of the 8544 did not have a phenotype described in OMIM. Of these 7412 genes:

- 1.3% had a RVIS percentile score < 10% (Fig. 4, yellow bar);
- None had a missense Z score > 6.23 (Fig. 4, yellow bar); and,
- 87 genes had a pLI score > 0.998 (1.2%) (Fig. 4, yellow bar), and 66 of the 87 genes had a pLI score = 1 (Fig. 4, yellow bar).

#### Genes published as a result of a GeneMatcher match

As of 12 June 2020, GeneMatcher was acknowledged in 325 publications and 302 genes were published in 249 publications acknowledging GeneMatcher-based collaborations. Two hundred twenty one of these genes (~ 73%) were in GeneMatcher for more than a year before publication (Fig. 4, green bar). Two hundred thirty one genes (~ 77%) were associated with a phenotype in OMIM, while 73 (~ 32%) had an OMIM phenotype before the gene was posted to GeneMatcher. Sixteen of the 302 genes (~ 5%) were also selected by Hansen et al. [21] as one of their 154 disease-causing candidate genes.

- 81 (~ 27%) had a RVIS percentile score of < 10% (Fig. 4, green bar).
- 6 (2%) had a missense Z score > 6.23 (Fig. 4, green bar).
- 93 (~ 31%) genes had a pLI score > 0.998 (Fig. 4, green bar); and 82 of these (27.2%) genes had a pLI score = 1 (Fig. 4, green bar).

Of the 302 published genes: 157 were associated with an autosomal dominant disease phenotype; 105 were associated with an autosomal recessive disease phenotype and biallelic variation in the proband; 24 were associated with both an autosomal dominant and autosomal recessive disease phenotype; and, 6 were associated with a X-linked phenotype.

### Future implementations

GeneMatcher modifications currently being implemented include connectivity to other relevant databases through the MME API and modifications that facilitate its use for model organism researchers.

### GeneMatcher experience

As part of the Baylor-Hopkins Center for Mendelian Genomics (BHCMG) project we submitted 768 unique candidate genes (1 June 2020). Of these, 651 genes had a match.

By following up on 413 of the 788 genes, we found that:

- 345 genes were matched
- For 33 genes, the gene matches resulted in a similar phenotype match between the BHCMG case and that of the other submitter; 30 of these genes were published (See Supplemental List 1)
- For 65 genes, gene matches resulted in a partial overlap of phenotypic features and are being further followed up in different ways
- Among the apparently negative matches:
   1—392 were between phenotypes with a different mode of inheritance;
   2—10 involved a false positive variant;
   3—3 involved the same patient;
   4—2 involved a variant that did not segregate with the phenotype in one of the families;
   5—163 involved genes that were entered in GeneMatcher >5 times and were associated with variable phenotype and variable zygosity of the candidate variants.

Of the 413 genes with follow up, 163 were entered between 5 and 151 times by unrelated investigators.

Of the 163 that were entered > 5 times, 133 were not associated with a phenotype in OMIM.

- 118 of the 133 genes (~ 17%) had RVIS %tile scores < 10%.
- One (0.8%) had missense Z scores > 6.23.
- 30 (~ 23%) had a pLI score > 0.998, and 26 of the 30 had a pLI score = 1 (20%).

Of the candidate variants in these 163 genes, 142 were rare (MAF < 1% in gnomAD) missense and 31 were rare loss-of-function variants (nonsense, splicing, frameshift indels). Twenty-one of these 163 genes were published as disease genes in the last 7 years (See Supplemental List 2).

### VariantMatcher

#### Introduction to VariantMatcher

VariantMatcher was developed to connect individuals (researchers, clinicians, and health care providers) around the globe with interest in a specific variant. It enables sharing of variant-level and phenotypic data from participants in research projects on the discovery of novel disease genes. VariantMatcher [22] contains the rare (MAF < 1%), coding (synonymous excluded), single nucleotide variants identified in 6,151 VCF files (897,720 unique variants) of affected and unaffected individuals sequenced as part of multiple projects and their phenotypic information.

To comply with patient privacy and security regulations, users of the site must register and be approved by site administrators. Users may upload up to 10 genomic coordinates per day to the site and are notified of any match. The query format is “chr:coordinate refAllele > altAllele” (e.g. chr2:1234567G > T) and is available for genomic builds hg18, hg19, or hGRC38. The current Mendelian Genomics PhenoDB data is on the hg19 build of the human reference assembly. Queries that use hg18 or hg38 are lifted over to hg19 before the match occurs. Phenotypic features can also be added but the match is based only on the genomic location. If the user adds phenotype information (minimum of three features and maximum of six), however, and there is a match based on genomic location, the phenotype information from the matched entries is shared in the email notifying the user of the match. When there is a match, both parties are notified by simultaneous emails so that they can choose to exchange additional information about their cases. If a match is not made, the queried coordinates can be stored for future matching. As of 1 April 2021, VariantMatcher had 642 submitters from 43 countries and 67 queried variants matched to 803 individuals.

#### An analysis of the data stored in VariantMatcher (data freeze 1 December 2020)

VariantMatcher had 611 users from 43 countries and 60 variants matched (of 428 variants that were queried) to 595 individuals in the database.

The 60 variants were observed in 56 genes, 40 of which were known disease-causing genes: 10 inherited as autosomal dominant phenotypes, 15 inherited as autosomal recessive phenotypes, 4 inherited as X-linked phenotypes, 1 inherited as a Y-linked phenotype, and 10 causing phenotypes with multiple modes of inheritance. The MAF of the 60 variants ranged from 0 to 0.44 but most (~ 97%) had a MAF < 0.1% in gnomAD. To date, no variant match was confirmed as a phenotype match but many matches were useful to potentially rule out a candidate variant. In the cases where the variant matched but the individuals’ phenotypes were different:

1. the VariantMatcher user had a patient with a homozygous variant and the individuals in VariantMatcher were heterozygous (1 variant match);
2. the VariantMatcher user had a male patient with a hemizygous variant while the individuals in VariantMatcher were female heterozygotes (1 variant match);
3. the variant was most likely benign and matched to multiple affected and unaffected individuals in VariantMatcher with different phenotypes (40 variant matches); or,
4. the variant was most likely benign and matched to affected individual(s) in VariantMatcher with different phenotype(s) (18 variant matches).

### Future implementations

To further develop VariantMatcher’s capabilities to support variant classification and facilitate discovery of disease-causing variants, we plan additional capabilities including: (1) indels; (2) variants by zygosity state; (3) specific variant with feature(s); and (4) specific group of variants/gene (e.g., individuals with nonsense variants in gene X). We also plan to connect VariantMatcher to other variant-level databases.

## Discussion

PhenoDB is a freely available web-based system that allows storage and analysis of phenotypic and genomic data. Future implementations will enable analysis of CNVs and mitochondrial sequencing data, and easier integration with other genomic analysis platforms, such as AnVIL (https://anvilproject.org/) and CAVATIVA (https://cavatica.squarespace.com/).

GeneMatcher has enabled hundreds of collaborations, disease-gene discoveries, better delineation of rare Mendelian disease traits and more precise variant classification. It is a powerful tool used by research and clinical laboratories investigating the genetic basis of rare diseases around the world as described by Bruel et al. [23]. To understand better which genes are in GeneMatcher and which genes are classified as causative of Mendelian disease, we analyzed features such RVIS %tile scores, gnomAD missense Z scores and pLI scores [24] of the genes in GeneMatcher. We found that over half of the genes entered in the database > 5 times were predicted to be highly conserved and intolerant to variation by at least one of these metrics (Fig. 4). Additionally, 109 of the 154 genes selected by Hansen et al. [21] were also entered into GeneMatcher > 5 times. This was anticipated since evolutionary conservation scores make it more likely for these genes to be selected as candidate genes and therefore entered by multiple investigators in GeneMatcher. Meanwhile, 21 of the 163 BHCMG genes (~ 13%) that were entered > 5 times were determined to be novel disease-causing genes to date.

In contrast, the percentage of highly conserved and intolerant to variation genes among the genes that did not match yet in GeneMatcher was far smaller (Fig. 4) and only 54 of these 4,730 were associated with a phenotype in OMIM and only 2 of these associations occurred in the last seven years. Again, perhaps not unexpectedly, since less conserved in silico prediction scores make these genes less likely to be selected as candidate genes and thus less likely to be submitted to GeneMatcher. Do certain variants in these genes produce a disease phenotype? Perhaps, despite the less conserved scores, these genes may play a role in human disease due to: 1—particular variants in the gene; 2—oligogenic mode of inheritance, 3—polygenic mode of inheritance; and 4—imprinting mode of inheritance. Perhaps the phenotypes associated with these genes have not been identified because of a mild presentation, late disease onset or other reasons that currently evade our limited molecular understanding of penetrance and variability of expression. Hence, criteria other than in silico prediction scores, and alternative bioinformatic analyses will be needed to experimentally establish these genes as ‘disease associated genes’.

While a PubMed search identified 302 GeneMatcher genes published, the analysis of the genes that were entered > 5 times in GeneMatcher and that had an OMIM phenotype as of 1 June 2020, showed that 445 genes were associated with an OMIM phenotype after being deposited in GeneMatcher, suggesting that many more disease-gene discoveries were enabled by GeneMatcher.

Finally, VariantMatcher has been shown to be a useful tool for ruling out potential causative variants, although users should consider incomplete penetrance, variable expressivity of the phenotype, and age of onset when the phenotypes under investigation are being compared. We suggest that VariantMatcher will also increase novel disease gene discovery by increasing the specificity of matches and facilitating the reclassification of variants of uncertain significance.

## Conclusion

In summary, PhenoDB, GeneMatcher and VariantMatcher have facilitated data analysis, improved the ability to share data globally, and to connect genes to phenotypes through 1—collective analysis of sequencing data generated in multiple independent centers; 2—identification of multiple patients around the world with the same rare Mendelian diseases; 3—variant classification; and, 4—better definition of the phenotypic variability of rare Mendelian diseases. Challenges remain for both researchers and clinicians as explorations of human biology and medicine further seek to understand perturbations of biological homeostasis that can result in disease. Nevertheless, one clear path forward is to understand the genes and variants that contribute to our individuality.

## Supplementary Information

**Additional file 1**. Publication list of 30 genes from the BHCMG project published as a result of a successful GeneMatcher collaboration.

**Additional file 2**. Publication list of 21 genes from the BHCMG project which were published in the last 7 years from the 163 genes submitted to GeneMatcher ≥ 5 times

## Data Availability

Not applicable.

## Abbreviations

ES: Exome sequencing
GS: Genome sequencing
VCF: Variant call format
OMIM: Online Mendelian Inheritance in Man
MAF: Minor allele frequency
MGI: Mouse Genome Informatics
GWAS: Genome wide association study
RVIS: Residual Variation Intolerance Score
ACMG: American College of Medical Genetics
CIDR: Center for Inherited Disease Research
JHU: Johns Hopkins University
HGSC: Human Genome Sequencing Center
MME: Matchmaker Exchange
BAM: Binary alignment map
CRAM: Condensed file from binary alignment map
IGV: Integrative Genomics Viewer
CNV: Copy number variant
