# Supplementary material for: PhenoDB, GeneMatcher and VariantMatcher, tools for analysis and sharing of sequence data
Source: Orphanet J Rare Dis. 2021 Aug 18;16:365. doi: 10.1186/s13023-021-01916-z (PMC8371856; doi:10.1186/s13023-021-01916-z)
Supplement: Supplementary file 1 — Additional file 1. Publication list of 30 genes from the BHCMG project published as a result of a successful GeneMatcher collaboration. [file 13023_2021_1916_MOESM1_ESM.docx]

**Supplemental List 1:** Publication list of 30 genes from the BHCMG project published as a result of a successful GeneMatcher collaboration.

1 - Alodaib A, Sobreira N, Gold WA, Riley LG, Van Bergen NJ, Wilson MJ, Bennetts B, Thorburn DR, Boehm C, Christodoulou J. Whole-exome sequencing identifies novel variants in PNPT1 causing oxidative phosphorylation defects and severe multisystem disease. Eur. J. Hum. Genet. 2016 Jan;25(1):79-84. doi:10.1038/ejhg.2016.128. PubMed: 27759031.

2 - Au PYB, You J, Caluseriu O, Schwartzentruber J, Majewski J, Bernier FP, Ferguson M, Care for Rare Canada Consortium, Valle D, Parboosingh JS, Sobreira N, Innes AM, Kline AD. GeneMatcher aids in the identification of a new malformation syndrome with intellectual disability, unique facial dysmorphisms, and skeletal and connective tissue abnormalities caused by de novo variants in HNRNPK. Hum. Mutat. 2015 Oct;36(10):1009-1014. doi:10.1002/humu.22837. PubMed: 26173930.

3 - Ben-Salem S, Robbins SM, Lm Sobreira N, Lyon A, Al-Shamsi AM, Islam BK, Akawi NA, John A, Thachillath P, Al Hamed S, Valle D, Ali BR, Al-Gazali L. Defect in phosphoinositide signalling through a homozygous variant in PLCB3 causes a new form of spondylometaphyseal dysplasia with corneal dystrophy. J. Med. Genet. 2018 Feb;55(2):122-130. doi:10.1136/jmedgenet-2017-104827. PubMed: 29122926.

4 - Ben-Salem S, Sobreira N, Akawi NA, Al-Shamsi AM, John A, Pramathan T, Valle D, Ali BR, Al-Gazali L. Gonadal mosaicism in ARID1B gene causes intellectual disability and dysmorphic features in three siblings. Am. J. Med. Genet. A. 2016 Jan;170A(1):156-61. doi:10.1002/ajmg.a.37405. PubMed: 26395437.

5 - Carvalho DR, Medeiros JEG, Ribeiro DSM, Martins BJAF, Sobreira NLM. Additional features of Gillespie syndrome in two Brazilian siblings with a novel ITPR1 homozygous pathogenic variant. Eur J Med Genet. 2018 Mar;61(3):134-138. doi:10.1016/j.ejmg.2017.11.005. PubMed: 29169895.

6 - Castilla-Vallmanya L, Selmer KK, Dimartino C, Rabionet R, Blanco-Sánchez B, Yang S, Reijnders MRF, van Essen AJ, Oufadem M, Vigeland MD, Stadheim B, Houge G, Cox H, Kingston H, Clayton-Smith J, Innis JW, Iascone M, Cereda A, Gabbiadini S, Chung WK, Sanders V, Charrow J, Bryant E, Millichap J, Vitobello A, Thauvin C, Mau-Them FT, Faivre L, Lesca G, Labalme A, Rougeot C, Chatron N, Sanlaville D, Christensen KM, Kirby A, Lewandowski R, Gannaway R, Aly M, Lehman A, Clarke L, Graul-Neumann L, Zweier C, Lessel D, Lozic B, Aukrust I, Peretz R, Stratton R, Smol T, Dieux-Coëslier A, Meira J, Wohler E, Sobreira N, Beaver EM, Heeley J, Briere LC, High FA, Sweetser DA, Walker MA, Keegan CE, Jayakar P, Shinawi M, Kerstjens-Frederikse WS, Earl DL, Siu VM, Reesor E, Yao T, Hegele RA, Vaske OM, Rego S; Undiagnosed Diseases Network, Care4Rare Canada Consortium, Shapiro KA, Wong B, Gambello MJ, McDonald M, Karlowicz D, Colombo R, Serretti A, Pais L, O'Donnell-Luria A, Wray A, Sadedin S, Chong B, Tan TY, Christodoulou J, White SM, Slavotinek A, Barbouth D, Morel Swols D, Parisot M, Bole-Feysot C, Nitschké P, Pingault V, Munnich A, Cho MT, Cormier-Daire V, Balcells S, Lyonnet S, Grinberg D, Amiel J, Urreizti R, Gordon CT. Phenotypic spectrum and transcriptomic profile associated with germline variants in TRAF7. Genet Med. 2020 Jul;22(7):1215-1226. doi: 10.1038/s41436-020-0792-7. Epub 2020 May 7. PMID: 32376980.

7 - Chacón-Camacho OF, Sobreira N, You J, Piña-Aguilar RE, Villegas-Ruiz V, Zenteno JC. Exome sequencing identifies a de novo frameshift mutation in the imprinted gene ZDBF2 in a sporadic patient with Nasopalpebral Lipoma-coloboma syndrome. Am. J. Med. Genet. A. 2016 Jul;170(7):1934-7. doi:10.1002/ajmg.a.37683. PubMed: 27139419.

8 - Fichtman B, Harel T, Biran N, Zagairy F, Applegate CD, Salzberg Y, Gilboa T, Salah S, Shaag A, Simanovsky N, Ayoubieh H, Sobreira N, Punzi G, Pierri CL, Hamosh A, Elpeleg O, Harel A, Edvardson S. Pathogenic Variants in NUP214 Cause "Plugged" Nuclear Pore Channels and Acute Febrile Encephalopathy. Am. J. Hum. Genet. 2019 Jul 3;105(1):48-64. doi:10.1016/j.ajhg.2019.05.003. PubMed: 31178128.

9 - Giorgio E, Sirchia F, Bosco M, Sobreira NLM, Baylor-Hopkins Center for Mendelian Genomics, Grosso E, Brussino A, Brusco A. A novel case of Greenberg dysplasia and genotype-phenotype correlation analysis for LBR pathogenic variants: An instructive example of one gene-multiple phenotypes. Am. J. Med. Genet. A. 2019 Feb;179(2):306-311. doi:10.1002/ajmg.a.61000. PubMed: 30561119.

10 - Gowans LJJ, Cameron-Christie S, Slayton RL, Busch T, Romero-Bustillos M, Eliason S, Sweat M, Sobreira N, Yu W, Kantaputra PN, Wohler E, Adeyemo WL, Lachke SA, Anand D, Campbell C, Drummond BK, Markie DM, van Vuuren WJ, van Vuuren LJ, Casamassimo PS, Ettinger R, Owais A, van Staden I, Amendt BA, Adeyemo AA, Murray JC, Robertson SP, Butali A. Missense Pathogenic variants in KIF4A Affect Dental Morphogenesis Resulting in X-linked Taurodontism, Microdontia and Dens-Invaginatus. Front Genet. 2019;10:800. doi:10.3389/fgene.2019.00800. PubMed: 31616463.

11 - Jurgens J, Sobreira N, Modaff P, Reiser CA, Seo SH, Seong MW, Park SS, Kim OH, Cho TJ, Pauli RM. Novel COL2A1 variant (c.619G>A, p.Gly207Arg) manifesting as a phenotype similar to progressive pseudorheumatoid dysplasia and spondyloepiphyseal dysplasia, Stanescu type. Hum. Mutat. 2015 Oct;36(10):1004-8. doi:10.1002/humu.22839. PubMed: 26183434.

12 - Lee CS, Fu H, Baratang N, Rousseau J, Kumra H, Sutton VR, Niceta M, Ciolfi A, Yamamoto G, Bertola D, Marcelis CL, Lugtenberg D, Bartuli A, Kim C, Hoover-Fong J, Sobreira N, Pauli R, Bacino C, Krakow D, Parboosingh J, Yap P, Kariminejad A, McDonald MT, Aracena MI, Lausch E, Unger S, Superti-Furga A, Lu JT, Baylor-Hopkins Center for Mendelian Genomics, Cohn DH, Tartaglia M, Lee BH, Reinhardt DP, Campeau PM. Mutations in Fibronectin Cause a Subtype of Spondylometaphyseal Dysplasia with "Corner Fractures". Am. J. Hum. Genet. 2017 Nov 2;101(5):815-823. doi:10.1016/j.ajhg.2017.09.019. PubMed: 29100092.

13 - Lee-Barber J, English TE, Britton JF, Sobreira N, Goldstein J, Valle D, Bjornsson HT. Apparent Acetaminophen Toxicity in a Patient with Transaldolase Deficiency. JIMD Rep. 2019;44:9-15. doi:10.1007/8904_2018_116. PubMed: 29923087.

14 - Li R, Sobreira N, Witmer PD, Pratz KW, Braunstein EM. Two novel germline DDX41 mutations in a family with inherited myelodysplasia/acute myeloid leukemia. Haematologica. 2016 Jun;101(6):e228-31. doi:10.3324/haematol.2015.139790. PubMed: 26944477.

15 - Lu JG, Bishop J, Cheyette S, Zhulin IB, Guo S, Sobreira N, Brenner SE. A novel PRRT2 pathogenic variant in a family with paroxysmal kinesigenic dyskinesia and benign familial infantile seizures. Cold Spring Harb Mol Case Stud. 2018 Feb;4(1). doi:10.1101/mcs.a002287. PubMed: 29167286.

16 - Mealy MA, Nam TS, Pardo SJ, Pardo CA, Sobreira NL, Avramopoulos D, Valle D, Burns KH, Levy M. Familial monophasic acute transverse myelitis due to the pathogenic variant in VPS37A. Neurol Genet. 2018 Feb;4(1):e213. doi:10.1212/NXG.0000000000000213. PubMed: 29473047.

17 - Moreno CA, Metze K, Lomazi EA, Bertola DR, Barbosa RH, Cosentino V, Sobreira N, Cavalcanti DP. Visceral myopathy: Clinical and molecular survey of a cohort of seven new patients and state of the art of overlapping phenotypes. Am. J. Med. Genet. A. 2016 Nov;170(11):2965-2974. doi:10.1002/ajmg.a.37857. PubMed: 27481187.

18 - Pant DC, Dorboz I, Schluter A, Fourcade S, Launay N, Joya J, Aguilera-Albesa S, Yoldi ME, Casasnovas C, Willis MJ, Ruiz M, Ville D, Lesca G, Siquier-Pernet K, Desguerre I, Yan H, Wang J, Burmeister M, Brady L, Tarnopolsky M, Cornet C, Rubbini D, Terriente J, James KN, Musaev D, Zaki MS, Patterson MC, Lanpher BC, Klee EW, Pinto E Vairo F, Wohler E, Sobreira NLM, Cohen JS, Maroofian R, Galehdari H, Mazaheri N, Shariati G, Colleaux L, Rodriguez D, Gleeson JG, Pujades C, Fatemi A, Boespflug-Tanguy O, Pujol A. Loss of the sphingolipid desaturase DEGS1 causes hypomyelinating leukodystrophy. J. Clin. Invest. 2019 Mar 1;129(3):1240-1256. doi:10.1172/JCI123959. PubMed: 30620337.

19 - Sisk RA, Hufnagel RB, Laham A, Wohler ES, Sobreira N, Ahmed ZM. Peripheral Cone Dystrophy: Expanded Clinical Spectrum, Multimodal and Ultrawide-Field Imaging, and Genomic Analysis. J Ophthalmol. 2018;2018:2984934. doi:10.1155/2018/2984934. PubMed: 30116628.

20 - Sobreira N, Brucato M, Zhang L, Ladd-Acosta C, Ongaco C, Romm J, Doheny KF, Mingroni-Netto RC, Bertola D, Kim CA, Perez AB, Melaragno MI, Valle D, Meloni VA, Bjornsson HT. Patients with a Kabuki syndrome phenotype demonstrate DNA methylation abnormalities. Eur. J. Hum. Genet. 2017 Dec;25(12):1335-1344. doi:10.1038/s41431-017-0023-0. PubMed: 29255178.

21 - Tanaka AJ, Cho MT, Millan F, Juusola J, Retterer K, Joshi C, Niyazov D, Garnica A, Gratz E, Deardorff M, Wilkins A, Ortiz-Gonzalez X, Mathews K, Panzer K, Brilstra E, van Gassen KL, Volker-Touw CM, van Binsbergen E, Sobreira N, Hamosh A, McKnight D, Monaghan KG, Chung WK. Mutations in SPATA5 Are Associated with Microcephaly, Intellectual Disability, Seizures, and Hearing Loss. Am. J. Hum. Genet. 2015 Sep 3;97(3):457-64. doi:10.1016/j.ajhg.2015.07.014. PubMed: 26299366.

22 - Telegrafi A, Webb BD, Robbins SM, Speck-Martins CE, FitzPatrick D, Fleming L, Redett R, Dufke A, Houge G, van Harssel JJT, Verloes A, Robles A, Manoli I, Engle EC, Moebius Syndrome Research Consortium, Jabs EW, Valle D, Carey J, Hoover-Fong JE, Sobreira NLM. Identification of STAC3 variants in non-Native American families with overlapping features of Carey-Fineman-Ziter syndrome and Moebius syndrome. Am. J. Med. Genet. A. 2017 Oct;173(10):2763-2771. doi:10.1002/ajmg.a.38375. PubMed: 28777491.

23 - You J, Sobreira NL, Gable DL, Jurgens J, Grange DK, Belnap N, Siniard A, Szelinger S, Schrauwen I, Richholt RF, Vallee SE, Dinulos MBP, Valle D, Armanios M, Hoover-Fong J. A Syndromic Intellectual Disability Disorder Caused by Variants in TELO2, a Gene Encoding a Component of the TTT Complex. Am. J. Hum. Genet. 2016 May 5;98(5):909-918. doi:10.1016/j.ajhg.2016.03.014. PubMed: 27132593.
