# Supplementary material for: PhenoDB, GeneMatcher and VariantMatcher, tools for analysis and sharing of sequence data
Source: Orphanet J Rare Dis. 2021 Aug 18;16:365. doi: 10.1186/s13023-021-01916-z (PMC8371856; doi:10.1186/s13023-021-01916-z)
Supplement: Supplementary file 2 — Additional file 2. Publication list of 21 genes from the BHCMG project which were published in the last 7 years from the 163 genes submitted to GeneMatcher ≥ 5 times [file 13023_2021_1916_MOESM2_ESM.docx]

**Supplemental List 2:** Publication list of 21 genes from the BHCMG project which were published in the last 7 years from the 163 genes submitted to GeneMatcher ≥5 times.

1 - Castilla-Vallmanya L, Selmer KK, Dimartino C, Rabionet R, Blanco-Sánchez B, Yang S, Reijnders MRF, van Essen AJ, Oufadem M, Vigeland MD, Stadheim B, Houge G, Cox H, Kingston H, Clayton-Smith J, Innis JW, Iascone M, Cereda A, Gabbiadini S, Chung WK, Sanders V, Charrow J, Bryant E, Millichap J, Vitobello A, Thauvin C, Mau-Them FT, Faivre L, Lesca G, Labalme A, Rougeot C, Chatron N, Sanlaville D, Christensen KM, Kirby A, Lewandowski R, Gannaway R, Aly M, Lehman A, Clarke L, Graul-Neumann L, Zweier C, Lessel D, Lozic B, Aukrust I, Peretz R, Stratton R, Smol T, Dieux-Coëslier A, Meira J, Wohler E, Sobreira N, Beaver EM, Heeley J, Briere LC, High FA, Sweetser DA, Walker MA, Keegan CE, Jayakar P, Shinawi M, Kerstjens-Frederikse WS, Earl DL, Siu VM, Reesor E, Yao T, Hegele RA, Vaske OM, Rego S; Undiagnosed Diseases Network, Care4Rare Canada Consortium, Shapiro KA, Wong B, Gambello MJ, McDonald M, Karlowicz D, Colombo R, Serretti A, Pais L, O'Donnell-Luria A, Wray A, Sadedin S, Chong B, Tan TY, Christodoulou J, White SM, Slavotinek A, Barbouth D, Morel Swols D, Parisot M, Bole-Feysot C, Nitschké P, Pingault V, Munnich A, Cho MT, Cormier-Daire V, Balcells S, Lyonnet S, Grinberg D, Amiel J, Urreizti R, Gordon CT. Phenotypic spectrum and transcriptomic profile associated with germline variants in TRAF7. Genet Med. 2020 Jul;22(7):1215-1226. doi: 10.1038/s41436-020-0792-7. Epub 2020 May 7. PMID: 32376980.

2 - Chacón-Camacho OF, Sobreira N, You J, Piña-Aguilar RE, Villegas-Ruiz V, Zenteno JC. Exome sequencing identifies a de novo frameshift mutation in the imprinted gene ZDBF2 in a sporadic patient with Nasopalpebral Lipoma-coloboma syndrome. Am. J. Med. Genet. A. 2016 Jul;170(7):1934-7. doi:10.1002/ajmg.a.37683. PubMed: 27139419.

3 - Duijkers FA, McDonald A, Janssens GE, Lezzerini M, Jongejan A, van Koningsbruggen S, Leeuwenburgh-Pronk WG, Wlodarski MW, Moutton S, Tran-Mau-Them F, Thauvin-Robinet C, Faivre L, Monaghan KG, Smol T, Boute-Benejean O, Ladda RL, Sell SL, Bruel AL, Houtkooper RH, MacInnes AW. HNRNPR Variants that Impair Homeobox Gene Expression Drive Developmental Disorders in Humans. Am. J. Hum. Genet. 2019 Jun 6;104(6):1040-1059. doi:10.1016/j.ajhg.2019.03.024. PubMed: 31079900.

4 - Gabriele M, Vulto-van Silfhout AT, Germain PL, Vitriolo A, Kumar R, Douglas E, Haan E, Kosaki K, Takenouchi T, Rauch A, Steindl K, Frengen E, Misceo D, Pedurupillay CRJ, Stromme P, Rosenfeld JA, Shao Y, Craigen WJ, Schaaf CP, Rodriguez-Buritica D, Farach L, Friedman J, Thulin P, McLean SD, Nugent KM, Morton J, Nicholl J, Andrieux J, Stray-Pedersen A, Chambon P, Patrier S, Lynch SA, Kjaergaard S, Tørring PM, Brasch-Andersen C, Ronan A, van Haeringen A, Anderson PJ, Powis Z, Brunner HG, Pfundt R, Schuurs-Hoeijmakers JHM, van Bon BWM, Lelieveld S, Gilissen C, Nillesen WM, Vissers LELM, Gecz J, Koolen DA, Testa G, de Vries BBA. YY1 Haploinsufficiency Causes an Intellectual Disability Syndrome Featuring Transcriptional and Chromatin Dysfunction. Am. J. Hum. Genet. 2017 Jun 1;100(6):907-925. doi:10.1016/j.ajhg.2017.05.006. PubMed: 28575647.

5 - Karakaya M, Mazaheri N, Polat I, Bharucha-Goebel D, Donkervoort S, Maroofian R, Shariati G, Hoelker I, Monaghan K, Winchester S, Zori R, Galehdari H, Bönnemann CG, Yis U, Wirth B. Biallelic MCM3AP mutations cause Charcot-Marie-Tooth neuropathy with variable clinical presentation. Brain. 2017 Oct 1;140(10):e65. doi:10.1093/brain/awx222. PubMed: 28969388.

6 - Oud MM, Tuijnenburg P, Hempel M, van Vlies N, Ren Z, Ferdinandusse S, Jansen MH, Santer R, Johannsen J, Bacchelli C, Alders M, Li R, Davies R, Dupuis L, Cale CM, Wanders RJA, Pals ST, Ocaka L, James C, Müller I, Lehmberg K, Strom T, Engels H, Williams HJ, Beales P, Roepman R, Dias P, Brunner HG, Cobben JM, Hall C, Hartley T, Le Quesne Stabej P, Mendoza-Londono R, Davies EG, de Sousa SB, Lessel D, Arts HH, Kuijpers TW. Mutations in EXTL3 Cause Neuro-immuno-skeletal Dysplasia Syndrome. Am. J. Hum. Genet. 2017 Feb 2;100(2):281-296. doi:10.1016/j.ajhg.2017.01.013. PubMed: 28132690.

7 - Pant DC, Dorboz I, Schluter A, Fourcade S, Launay N, Joya J, Aguilera-Albesa S, Yoldi ME, Casasnovas C, Willis MJ, Ruiz M, Ville D, Lesca G, Siquier-Pernet K, Desguerre I, Yan H, Wang J, Burmeister M, Brady L, Tarnopolsky M, Cornet C, Rubbini D, Terriente J, James KN, Musaev D, Zaki MS, Patterson MC, Lanpher BC, Klee EW, Pinto E Vairo F, Wohler E, Sobreira NLM, Cohen JS, Maroofian R, Galehdari H, Mazaheri N, Shariati G, Colleaux L, Rodriguez D, Gleeson JG, Pujades C, Fatemi A, Boespflug-Tanguy O, Pujol A. Loss of the sphingolipid desaturase DEGS1 causes hypomyelinating leukodystrophy. J. Clin. Invest. 2019 Mar 1;129(3):1240-1256. doi:10.1172/JCI123959. PubMed: 30620337.
